# Evaluation of MRI Features and Neurodevelopmental Outcomes for Prenatally Diagnosed Periventricular Pseudocysts

**DOI:** 10.3389/fped.2021.681999

**Published:** 2021-10-22

**Authors:** Cong Sun, Xinjuan Zhang, Xin Chen, Xinhong Wei, Yufan Chen, Aocai Yang, Jinxia Zhu, Guangbin Wang

**Affiliations:** ^1^Department of Radiology, Cheeloo College of Medicine, Shandong Provincial Hospital, Shandong University, Jinan, China; ^2^Department of Radiology, Shandong Provincial Hospital Affiliated to Shandong First Medical University, Jinan, China; ^3^Department of Ultrasound, Shandong Provincial Hospital Affiliated to Shandong First Medical University, Jinan, China; ^4^MR Collaboration, Siemens Healthcare Ltd., Beijing, China

**Keywords:** fetus, periventricular pseudocyst, magnetic resonance imaging, morphological features, neurodevelopmental outcome

## Abstract

**Objectives:** This study aimed to evaluate the morphologic features and neurodevelopmental outcomes of individuals prenatally diagnosed with a periventricular pseudocyst (PVPC).

**Methods:** Pregnant women with a fetus prenatally diagnosed with PVPC by MRI were enrolled in this retrospective study. The fetuses with PVPCs were divided into group 1 (isolated PVPC) and group 2 (PVPC with additional findings). The surviving infants underwent brain MRI examinations and the Gesell Developmental Scale (GDS) test after birth. Independent sample *t*-tests were used to compare the differences in the developmental quotient (DQ) between group 1 and group 2. We also analyzed the correlations among the DQ, location (unilateral/bilateral), size (diameter), and number (single/multiple) of the PVPCs in group 1 using Lasso regression.

**Results:** In total, 131 infants (group 1: 78 infants, group 2: 53 infants) underwent MRI examinations after birth, and 97 infants (group 1: 59 infants, group 2: 38 infants) underwent the GDS test. Upon follow-up, the sizes of the cysts had become smaller or disappeared after birth. The average DQ in group 2 was lower than that in group 1 (all with *p* < 0.001). In group 1, the location (unilateral/bilateral), size (diameter), and number (single/multiple) of the PVPC did not affect the DQ.

**Conclusions:** The PVPCs became smaller or disappeared after birth. Isolated PVPCs usually have a normal presentation after birth regardless of the location, number, or size. For PVPCs with additional findings, the neurodevelopmental outcomes were inferior to those in isolated PVPCs.

## Introduction

Periventricular pseudocysts (PVPCs) are cystic cavities that lack the ependymal cell lining found in true cysts (1–4). PVPCs are thought to be the result of antenatal cystic germinal matrix regression or congenital viral infection, hemorrhage, or infarction (2, 5, 6). With improvements in ultrasound (US) and the wide use of fetal MRI, the detection rate of fetal PVPCs has gradually increased. By using US in the first few days of life, Heibel et al. found that PVPCs occurred in 0.5–5% of healthy term neonates (7). This presents a challenge for antenatal consultation and can lead to anxiety for pregnant women. In addition, many radiologists often confuse the cystic lesions seen in PVPCs and periventricular leukomalacia (PVL); differentiation between the two conditions is facilitated by noting their distinct anatomic locations (PVPC: below the superolateral angles of the frontal horns or body of the lateral ventricles; and PVL: above the superolateral angles of the frontal horns) (2). Therefore, it is critical to explore the morphological features, alterations, and neurodevelopmental outcomes of PVPCs before and after birth.

To date, some studies have focused on the neurodevelopmental outcomes of PVPCs, but uncertainty still exists regarding their clinical significance (8–10). Esteban et al. focused on the prenatal features of isolated subependymal pseudocysts and suggested that a PVPC great axis over 9 mm should raise suspicion for underlying pathology (11). In contrast, Yang et al. also addressed the diagnosis and prognosis of isolated PVPCs, but concluded that the size and number do not affect the outcome of unilateral cysts (12). Wang et al. found that isolated PVPCs did not affect white matter microstructure development at the neonatal stage (13). However, these published studies included small sample sizes and limited subject follow-up (6, 8, 10). To our knowledge, almost all of these studies focused only on the US characteristics of the PVPCs; the MRI imaging features of PVPCs with large sample sizes are lacking (1, 9, 14, 15).

Therefore, the aims of this present study were as follows: (1) to explore the morphological characteristics of PVPCs before and after birth using MRI; (2) to compare the neurodevelopmental outcomes of both isolated PVPCs and those with additional findings; and (3) to investigate the correlation between morphological features of isolated PVPCs and neurodevelopmental outcome.

## Materials and Methods

### Patients

This study was approved by our Institutional Review Board, and written informed consent was obtained from all the patients. Between June 2015 and October 2019, pregnant women with fetuses who were prenatally diagnosed with PVPCs by MRI in our center were enrolled in this retrospective study. The exclusion criteria included: fetal PVL cysts diagnosed on antenatal MRI; fetuses without postnatal MRI follow-up results (for the morphological alterations of PVPCs); and fetuses without Gesell Developmental Scale (GDS) test results (assessing for the neurodevelopmental outcomes of PVPCs). Based on associations with intracranial or extracranial malformations, the patients were divided into group 1 (isolated PVPCs) and group 2 (PVPCs with additional findings). For the isolated PVPCs, no additional fetal abnormalities were found on prenatal MRI, and there was no fetal infection or chromosomal abnormalities. The “additional findings” were defined as the presence of other extra-intracranial abnormalities in the fetus, in addition to PVPCs. For the pregnant women, the following clinical data were recorded: age, gestational age for initial diagnosis of PVPCs, and gestational age at delivery. For the fetuses, the MR imaging features of the PVPCs and the details of the additional findings were recorded. For the neonatal data, the birth weight, Apgar scores, postnatal MRI follow-up age, and the GDS results were recorded.

### MRI Acquisition

The MRI examinations for the pregnant women were all performed on a 1.5 T system (MAGNETOM Amira, Siemens Magnetic Resonance, Ltd.) with a 13-channel body coil in combination with a 32-channel spine coil. The entire fetal body was included in the MR examination. All the pregnant women were imaged in the supine or left-lateral position. Half-Fourier single-shot turbo spin-echo (HASTE)/True fast imaging with steady-state precession (TrueFISP) and T1-weighted imaging (T1WI) images were acquired in the axial, coronal, and sagittal planes with the following parameters: HASTE: repetition time (TR) = 1,300 ms, echo time (TE) = 100 ms, field of view (FOV) = 380 × 309 mm, slice gap = 0, matrix = 256 × 198, voxel size = 1.5 × 1.5 × 3.5 mm^3^, and acquisition time = 28 s. TrueFISP: TR = 621.61 ms, TE = 1.76 ms, FOV = 380 × 310 mm, slice gap = 0, matrix = 304 × 199, voxel size = 1.3 × 1.3 × 4.0 mm^3^, and acquisition time = 12 s. T1WI: TR = 241 ms, TE = 4.62 ms, FOV = 380 × 322 mm, slice gap = 0, matrix = 256 × 162, voxel size = 1.5 × 1.5 × 3.5 mm^3^, and acquisition time = 18 s. No maternal sedation was used for the fetal MRI examinations, and specific absorption rate limits were consistent with departmental protocols.

Examinations of the infants/toddlers (6 months−3 years old) were all performed on a 3T system (MAGNETOM Skyra, Siemens Healthcare, Erlangen, Germany) with a 20-channel head array coil. We recorded the exact time when each postnatal MRI was obtained. All the pediatric patients were imaged in the supine position, and the imaging protocol included T1WI, T2-weighted imaging (T2WI) in the axial plane, and T2WI in the sagittal plane.

### Neurodevelopmental Outcome

After birth, all the infants underwent GDS testing between 0 and 3 years of age. The age at which the postnatal GDS test was performed was obtained in group 1 and group 2. The GDS is a classic international child development scale that examines five specific behaviors: adaptive, gross motor, fine motor, language, and personal social (16, 17). The results are expressed in terms of the developmental quotient (DQ), which is calculated as DQ = developmental age (DA)/actual age (CA) × 100. Children with a DQ ≥ 85 were deemed normal in terms of developmental status. Those with a DQ < 40 were diagnosed as having severe neurodevelopmental disabilities. Based on the degree of neurodevelopmental disability, the DQ scores were divided into mild (55 ≤ DQ < 75), moderate (40 ≤ DQ < 55), severe (25 ≤ DQ < 40), and suspected (75 ≤ DQ < 84) neurodevelopmental disability.

### Imaging Features

The fetal and infant MRIs were evaluated by an expert MR imaging neuroradiologist (W., with 15 years of experience in fetal brain imaging) and an obstetrician who specializes in fetal MR imaging (S., with 10 years of experience). The evaluated imaging characteristics of PVPCs included the following: location (unilateral or bilateral), size (the mean anteroposterior diameter and mean height), number (single/multiple), and size changes of the cysts before and after birth.

### Statistical Analysis

Data were presented as means ± standard deviations (SD) or medians and range for continuous data and numbers (percentages) for categorical data. The intraclass correlation coefficient (ICC) was used to analyze the consistency between the two reviewers. The independent-sample *t*-test was used to compare the exact time when postnatal MRI and GDS tests were obtained in group 1 and group 2. SPSS 22.0 (IBM Corp., Armonk/NY, USA) was used to perform the statistical analysis.

The correlations among the DQ, the location (unilateral/bilateral), size (the mean anteroposterior diameter and mean height), and the number (single/multiple) of PVPCs in group 1 were compared using Lasso regression, in which the average DQ of the adaptive, gross motor, fine motor, language, and personal social behaviors were taken as dependent variables, whereas the variables of location, size, and number of PVPCs were deemed to be independent variables. DQ was used to remove unimportant variables from the original data and focus on the selection of the most useful morphologic features of isolated PVPCs that affect the DQ. The adjustment parameter (lambda) in the Lasso model was chosen to pass the 10-fold cross-validation through the minimum standard. Lasso regression was conducted using Package “glmnet” in R (R Core Team, 2017, http://www.R-project.org). All the statistical tests were two-tailed, and *P* < 0.05 was considered statistically significant.

## Results

### Population Characteristics

**Figure 1:**
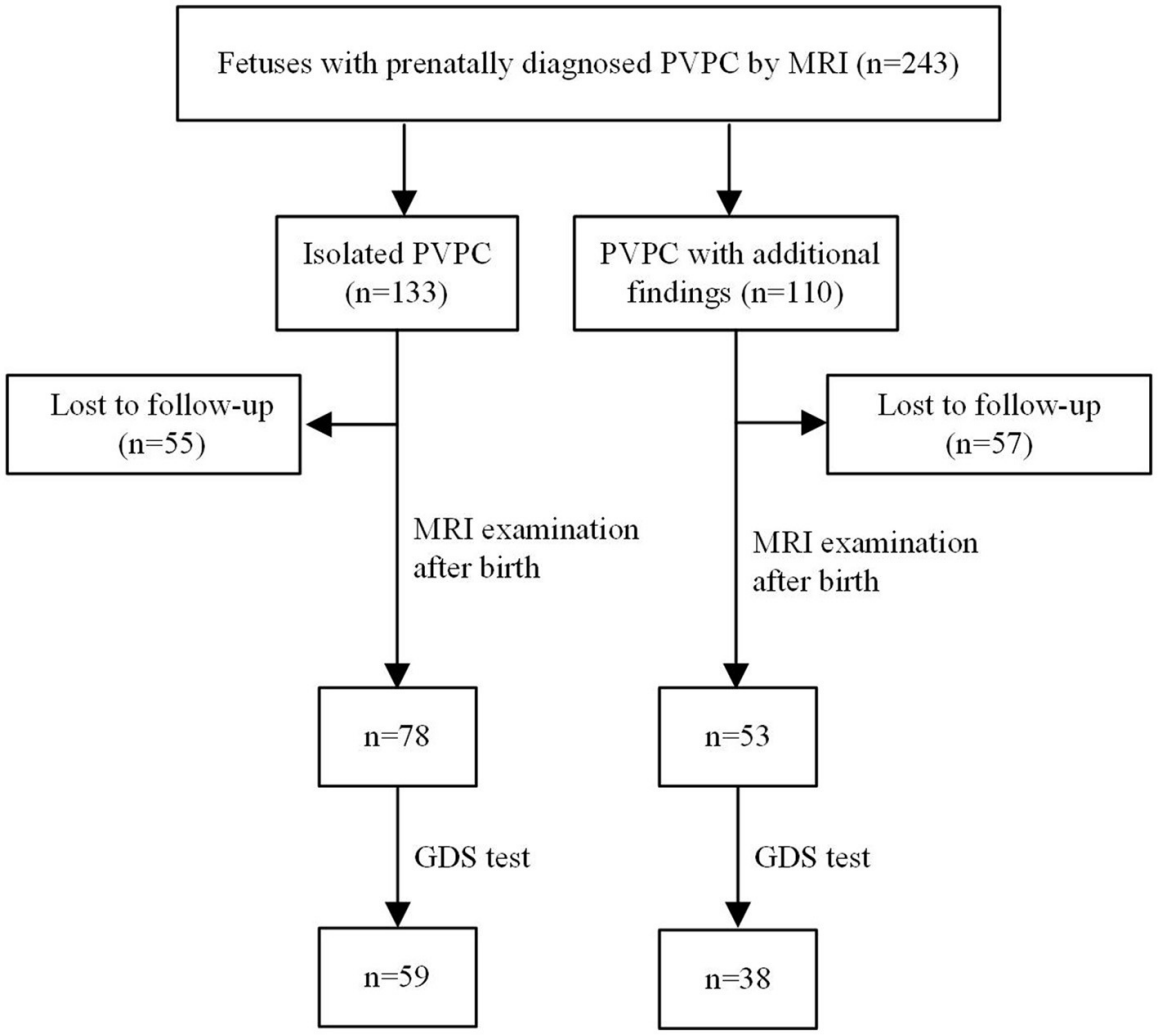
Patients and flow chart of study design. PVPC, periventricular pseudocyst; GDS, gesell developmental scale.

In total, 243 subjects with PVPCs prenatally diagnosed by MRI were enrolled. Among these 243 subjects, 133 (54.7%) had isolated PVPCs, and 110 (45.3%) had PVPCs with additional findings. Fifty-five (41.4%) fetuses in group 1 and 57 (51.8%) fetuses in group 2 were lost to follow-up because the pregnant women refused to have their infants undergo MRI examinations. Among the remaining 78 subjects in group 1 and 53 subjects in group 2, 34 subjects did not undergo the GDS test. Ultimately, 78 subjects in group 1 and 53 subjects in group 2 underwent postnatal MRI evaluation; 59 subjects in group 1 and 38 subjects in group 2 underwent a postnatal neurodevelopmental outcome evaluation. The mean gestational age for the initial diagnosis of PVPCs was 33 ± 4 weeks (range, 23–39 weeks). The patients and the flow chart of the study design are shown in Figure 1. The additional findings of group 2 included lateral ventriculomegaly, mega cisterna magna (MCM), arachnoid cyst, agenesis of the corpus callosum (ACC), leukodystrophy, cerebral hemorrhage, Dandy-Walker syndrome, nodular sclerosis, gray matter heterotopia, pachygyria, hypoxic-ischemic encephalopathy (HIE), TORCH virus infections, and chromosomal abnormality [microdeletion: del(11) (p15.4)]. The population characteristics are summarized in Table 1.

**Table 1:** Population characteristics.

**Characteristics**	**Mean ± standard deviation or proportion**
**Pregnant women**	
Age (y)	31 ± 5 (20~43)
Gestational age for initial diagnosis of PVPC (w)	33 ± 4 (23–39)
Gestational age at delivery (w)	38 ± 2 (36–41)
**Fetal and infant MRI**	
Isolated PVPC (n)	78 (59.5%)
PVPC with additional findings (n)	53 (40.5%)
Lateral ventriculomegaly (n)	18/53 (34.0%)
Mega cisterna magna (MCM) (n)	13/53 (24.5%)
Arachnoid cyst (n)	5/53 (9.43%)
Agenesis of the Corpus Callosum (ACC) (n)	4/53 (7.55%)
Leukodystrophy (n)	2/53 (3.77%)
Cerebral hemorrhage (n)	3/53 (5.66%)
Dandy-Walker syndrome (n)	1/53 (1.89%)
Nodular sclerosis (n)	1/53 (1.89%)
Gray matter heterotopia (n)	1/53 (1.89%)
Pachygyria (n)	1/53 (1.89%)
Hypoxic-ischemic encephalopathy (HIE)	2/53 (3.77%)
TORCH virus infections (n)	1/53 (1.89%)
Chromosomal abnormality (n)	1/53 (1.89%)
**Neonatal and infant data**	
Birth weight (g)	3,300 (2,400–4,200)
Apgar scores (5 min) (score)	9.7 (9–10)
The age at MRI examination after birth (m)	12 ± 4.5
Isolated PVPC GDS assessment (n)	59/78 (75.6%)
PVPC with additional findings GDS assessment (n)	38/53 (71.7%)
The age at GDS assessment (m)	13 ± 5 (6–30)

*Data are presented as means ± standard deviation (SD) or medians and range for continuous data and numbers (percentages) for categorical data*.

### MRI Features of Fetal PVPCs and Postnatal Follow-Up

**Figure 2:**
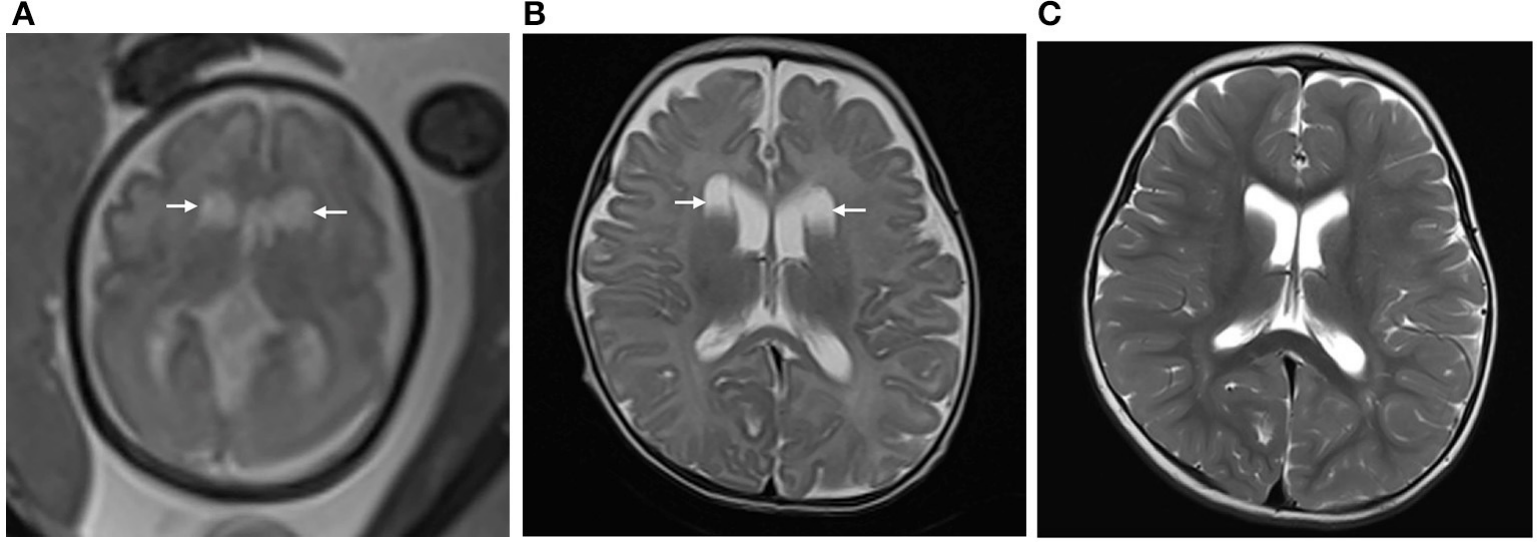
**(A)** Pregnant woman, 29 years old, with a fetus of 30 weeks' gestation. Fetal brain MRI shows bilateral isolated periventricular pseudocyst (PVPC) located at the external angle of the lateral ventricles (white arrow). The anteroposterior diameter is 1.5 and 1.6 cm, respectively; **(B)** 40 days after birth, the cyst sizes show no significant change; and **(C)** At the age of 18 months, the PVPCs disappeared, and the Gesell Developmental Scale (GDS) test showed the developmental quotient (DQ: Adaptive: 96, Gross Motor: 87, Fine Motor: 89, Language: 96, and Personal-Social: 91) was normal.

**Figure 3:**
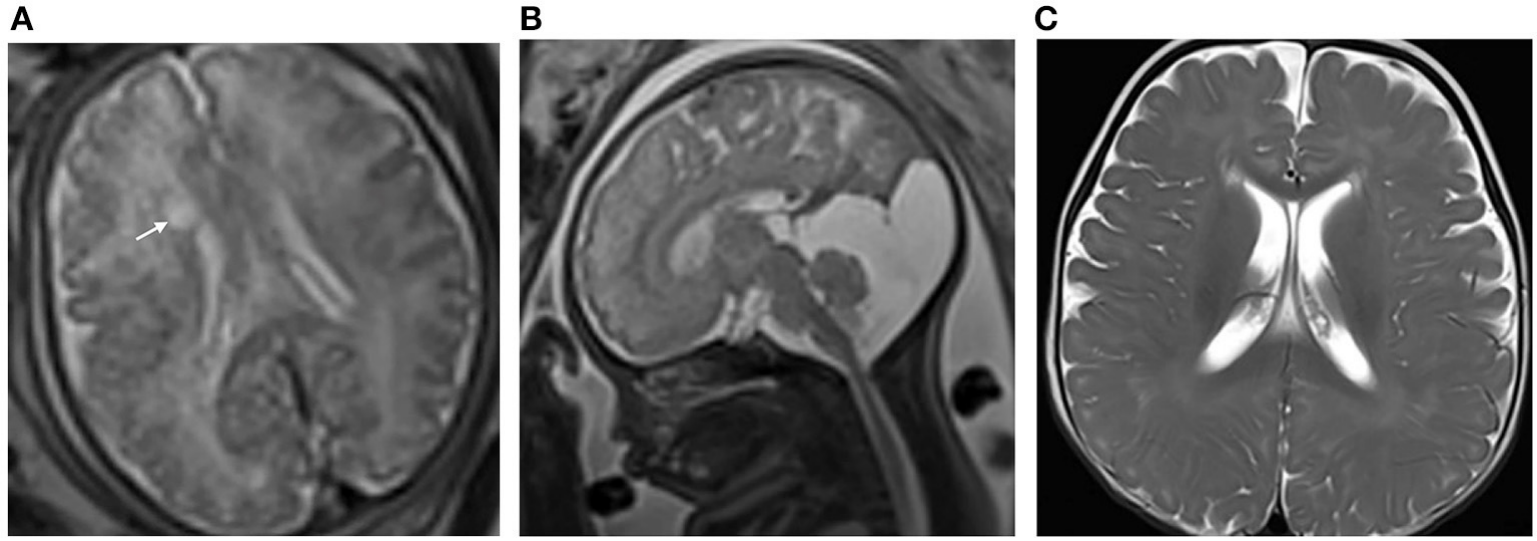
**(A)** Pregnant woman, 32 years old, with a fetus of 38 weeks' gestation. Fetal brain MRI shows a unilateral isolated periventricular pseudocyst (PVPC) located at the external angle of the lateral ventricle (white arrow); the anteroposterior diameter and height are 0.8 and 0.4 cm, respectively; **(B)** The additional finding is the large cyst in the cisterna magna (sagittal image); and **(C)** Female, 9 months after birth. The PVPC disappeared, and the Gesell Developmental Scale (GDS) test and developmental quotient showed a mild neurodevelopmental disability (DQ: Adaptive: 73, Gross Motor: 58, Fine Motor: 70, Language: 69, and Personal Social: 66).

Interobserver agreements between the two reviewers were good for the collected data (all ICC > 0.75). The MRI features and morphology alterations of the PVPCs before and after birth are summarized in Table 2. The age of postnatal MRI showed no difference between group 1 and group 2 (group 1: 12 ± 4 months, group 2: 12 ± 5 months, *p* > 0.05). Based on MRI follow-up, group 1 included subjects with 78 PVPCs, of which 56 disappeared (71.8%) (Figure 2), 15 decreased in size (19.2%), and seven showed no apparent changes in size (9.0%); in addition, no PVPCs enlarged after birth. Group 2 comprised 53 subjects; PVPCs disappeared in 36 of these subjects (67.9%) (Figure 3), decreased in size in 12 (22.6%), and remained stable in size in five of these subjects (9.4%); in addition, no PVPC enlarged after birth. The unilateral/bilateral (*p* = 0.705), mean anteroposterior diameter (*p* = 0.156), mean height (*p* = 0.221), single/multiple (*p* = 0.542) and the size changes of the PVPC after birth (*p* = 0.881) showed no difference between group 1 and group 2.

**Table 2:** MRI features and morphologic alterations of the PVPCs before and after birth.

**MRI features**	**Group 1**	**Group 2**	***P*-value**
	**(*n* = 78)**	**(*n* = 53)**	
Unilateral (left)	17 (21.8%)	15 (28.3%)	0.705
Unilateral (right)	8 (10.3%)	5 (9.43%)	
Bilateral (n)	53 (67.9%)	33 (62.3%)	
Mean anteroposterior diameter (cm)	1.56 ± 0.41	1.71 ± 0.65	0.156
Mean height (cm)	0.6 ± 0.19	0.75 ± 0.10	0.221
Single (n)	60 (76.9)	38 (71.7)	0.542
Multiple (n)	18 (23.1)	15 (28.3)	
**Cyst size change on the MRI**			
Enlargement (n)	0	0	0.881
No significant change (n)	7 (8.97%)	5 (9.43%)	
Smaller (n)	15 (19.2%)	12 (22.6%)	
Disappeared (n)	56 (71.8%)	36 (67.9%)	

*Data with normal distribution are represented as means ± standard deviation, and counting data are represented as n (percentage, %)*.

### Neurodevelopmental Outcomes

**Figure 4:**
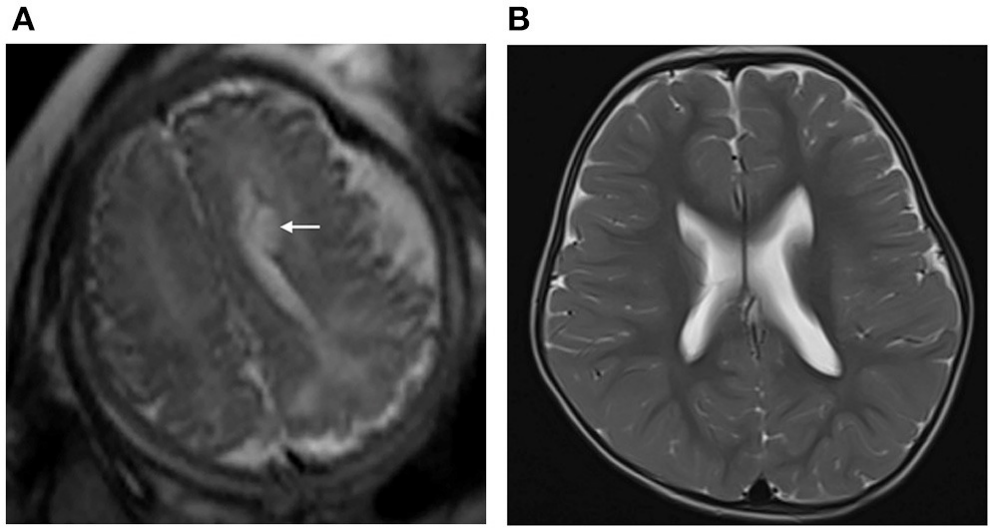
**(A)** Pregnant woman, 22 years old, with a fetus of 36 weeks' gestation. Fetal brain MRI shows a unilateral isolated periventricular pseudocyst (PVPC) located at the external angle of the lateral ventricle (white arrow); the anteroposterior diameter and height are 0.9 and 0.5 cm, respectively. **(B)** Female infant, with a history of hypoxic-ischemic encephalopathy (HIE) and asphyxia at birth. At the age of 30 months, the brain MRI showed the PVPC disappeared, and there were no changes in the thalami and basal ganglia related to the previous HIE. The GDS test showed a moderate neurodevelopmental disability in the Adaptive, Gross Motor, and Fine Motor parameters, and mild neurodevelopmental disability in the Language and Personal-Social parameters (DQ:Adaptive: 46, Gross Motor: 50, Fine Motor: 40, Language: 63, and Personal Social: 62).

The ages at GDS assessment after birth showed no difference between group 1 and group 2 (group 1: 13 ± 4 months, group 2: 13 ± 5 months, *p* > 0.05). The details of the neurodevelopmental outcomes of the PVPCs are shown in Table 3. Group 1 included 59 subjects, all of whom had a normal developmental outcome. Group 2 included 38 subjects, of whom four had a normal developmental outcome, 26 were suspected of having a neurodevelopmental disability, seven had a mild neurodevelopmental disability, and one was diagnosed with moderate neurodevelopmental disability. The average DQ of the adaptive, gross motor, fine motor, language, and personal social parameters in group 2 were all lower than those in group 1. The independent sample *t*-tests showed a significant difference in the DQ between the two groups, all with *p* < 0.01. Figure 4 shows a subject with a PVPC associated with HIE; the DQ was significantly lower than normal, and the subject was diagnosed with a moderate neurodevelopmental disability.

**Table 3:** Neurodevelopmental outcome of isolated PVPCs and PVPCs with additional findings.

**Development quotient**	**Group 1 (*n* = 59)**	**Group 2 (*n* = 38)**	***P*-value**
Adaptative	94.1 ± 4.7	77.0 ± 8.5	<0.001
Gross motor	91.8 ± 4.6	75.3 ± 7.4	<0.001
Fine motor	91.8 ± 6.0	74.7 ± 8.6	<0.001
Language	91.0 ± 4.1	76.4 ± 7.8	<0.001
Personal social	91.8 ± 4.5	75.2 ± 7.9	<0.001

*Data are represented as means ± standard deviation*.

*PVPC, Periventricular pseudocys*.

### Morphological Features of Isolated PVPC and Neurodevelopmental Outcome

**Figure 5:**
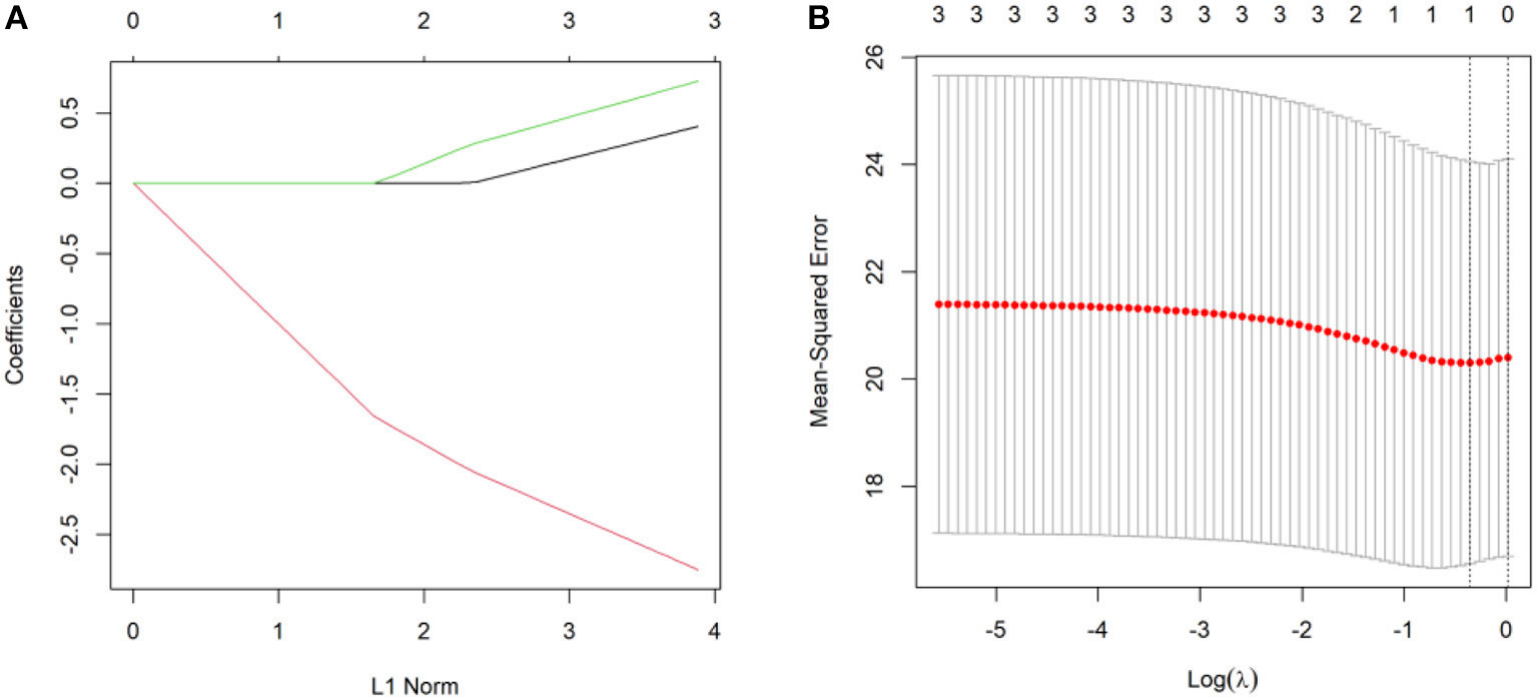
**(A)** A Lasso coefficient curve for three independent variables. **(B)** A logarithmic (lambda) sequence coefficient distribution.

Figure 5A shows the Lasso coefficient curve for three independent variables. Figure 5B shows a graph of the distribution of coefficients for a log (lambda) sequence. Vertical lines were drawn at the values selected using 10-fold cross-validation. The best lambda resulted in no non-zero coefficients, indicating that the location (unilateral/bilateral), size (the mean anteroposterior diameter and mean height), and the number (single/multiple) of the PVPCs did not affect the DQ.

## Discussion

In this study, isolated PVPCs usually became smaller or disappeared after birth and had normal presentations, regardless of the location, number, and size. Regarding the PVPCs with additional findings, the cysts also became smaller or disappeared, but the neurodevelopmental outcomes were inferior to those of isolated PVPCs.

Consistent with a previous study, the mean gestational age for the initial diagnosis of PVPCs in our cohort was also made in the third trimester (18). The percentage of bilateral PVPCs was much higher than the unilateral PVPCs, in both group1 and group 2. In addition, unilateral PVPCs were more often found on the left. This finding has also been observed in previous reports, and the reason for this is not clear, but it may be related to the differences in left- vs. right-side brain-blood circulation in the fetus (8, 12, 19). After birth, almost all the PVPCs regressed spontaneously in both group 1 and group 2. These results further support the idea that PVPCs may be a benign finding, and this is likely to be related to the mechanism of PVPC formation.

In our series, consistent with the literature, individuals in group 1 were all associated with a normal neurodevelopmental outcome (6, 8, 10, 12). However, the average DQ of the adaptive, gross motor, fine motor, language, and personal social parameters in group 2 were all lower than those in group 1. The significant difference in the DQ between the two groups may prove that the appearance of additional findings was the factor that significantly affected the postnatal outcome. However, the neurodevelopmental outcomes in group 2 were quite diverse and variable. With subjects who had PVPCs with Dandy-Walker syndrome and gray matter heterotopia, the DQ was significantly decreased compared to PVPCs with lateral ventriculomegaly and mega cisterna magna. These results prove that the DQ in group 2 could be closely related to the severity of the additional findings. However, we failed to make any further classifications because of the small sample size in each category. In the future, we plan to categorize the additional findings as this could lend further support to our premise that the PVPCs are benign.

Another important finding in our study was that the neurodevelopmental outcomes of isolated PVPCs were not related to the location (unilateral/bilateral), size, or number (single/multiple) of the PVPCs, and all had a good prognosis after birth. This finding differs from that in Estebani's study, which showed that cysts with a great axis ≥ 9 mm might be associated with adverse pregnancy outcomes (11). In addition, our finding is also contrary to those in previous studies which have suggested that bilateral multiple PVPC has a positive likelihood ratio of 9:1 for a chromosomal abnormality or a congenital infection in newborns (20). As a result, when prenatal MRIs find isolated PVPCs without additional inter-/extracranial abnormalities, we should not be overly concerned about the adverse prognosis, regardless of the location, size, or number of the cysts.

There were several limitations to this study. First, no fetal autopsies were performed to obtain precise pathological results. However, after birth, we followed up by performing postnatal imaging and clinical examinations on the infants. Second, because of the small sizes of some PVPCs or the thin walls being in close proximity to the ventricles, MRI might have failed to identify some multilocular cysts, so the proportion of single PVPCs might be higher. Third, because the cohort of connatal cysts was too small, we did not further classify the PVPCs into connatal cysts and subependymal cysts (2). Last, the age at which the neurodevelopmental outcome assessment was performed was soon after birth, and this time point could have been too early to allow recognition of cognitive outcome.

In conclusion, isolated PVPCs have a good clinical outcome, regardless of the cysts' location, size, and number. For PVPCs with additional findings, the neurodevelopmental outcomes were inferior to those in isolated PVPCs. Radiologists, pediatricians, and obstetricians should be aware of the imaging features and neurodevelopmental outcomes of PVPCs, which could offer useful information for antenatal consultation and predict neurodevelopmental outcomes in the fetal period.

## Data Availability Statement

The raw data supporting the conclusions of this article will be made available on request from the corresponding author.

## Ethics Statement

The studies involving human participants were reviewed and approved by Shandong Provincial Hospital Institutional Review Board. The patients/participants provided their written informed consent to participate in this study.

## Author Contributions

CS: conceptualization, visualization, methodology, resources, data curation, and writing—original draft. XZ: resources, visualization, methodology, data collection, and writing—review and editing. XC: resources, data curation, and funding acquisition. XW: visualization and statistical analysis. YC: data review and analysis. AY and JZ: writing—review and editing. GW: supervision, study design, project administration, and funding acquisition. All authors have critically reviewed, revised, and approved the manuscript in its final version.

## Funding

This work was supported by the National Natural Science Foundation of China [81671668] and the Natural Science Foundation of Shandong Province [ZR201911150560].

## Conflict of Interest

JZ was employed by the company Siemens Healthcare. The remaining authors declare that the research was conducted in the absence of any commercial or financial relationships that could be construed as a potential conflicts of interest.

## Publisher's Note

All claims expressed in this article are solely those of the authors and do not necessarily represent those of their affiliated organizations, or those of the publisher, the editors and the reviewers. Any product that may be evaluated in this article, or claim that may be made by its manufacturer, is not guaranteed or endorsed by the publisher.
